# Translational control plays a prominent role in the hepatocytic differentiation of HepaRG liver progenitor cells

**DOI:** 10.1186/gb-2008-9-1-r19

**Published:** 2008-01-25

**Authors:** Romain Parent, Laura Beretta

**Affiliations:** 1 Public Health Sciences Division, Fred Hutchinson Cancer Research Center, 1100 Fairview Avenue North (M5-A864), Seattle, Washington, 98109, USA

## Abstract

**Background:**

We investigated the molecular events associated with the differentiation of liver progenitor cells into functional and polarized hepatocytes, using human HepaRG cells that display potent hepatocytic differentiation-inducible properties and share some features with liver progenitor cells.

**Results:**

Profiling of total and of polysome-bound transcripts isolated from HepaRG cells undergoing hepatocytic differentiation was performed. A group of 3,071 probe sets was reproducibly regulated by at least 2-fold in total or in polysome-bound RNA populations, upon differentiation. The fold changes in the total and the polysome-bound RNA populations for these 3,071 probe sets were poorly correlated (R = 0.38). Moreover, while the majority of the regulated polysome-bound RNA probe sets were up-regulated upon differentiation, the majority of the regulated probe sets selected from the total RNA population was down-regulated. Genes translationally up-regulated were associated with cell cycle inhibition, increased susceptibility to apoptosis and innate immunity. In contrast, genes transcriptionally up-regulated during differentiation corresponded in the majority to liver-enriched transcripts involved in lipid homeostasis and drug metabolism. Finally, several epithelial and hepato-specific transcripts were strongly induced in the total RNA population but were translationally repressed.

**Conclusion:**

Translational regulation is the main genomic event associated with hepatocytic differentiation of liver progenitor cells *in vitro *and targets genes critical for moderating hepatocellular growth, cell death and susceptibility to pathogens. Transcriptional regulation targets specifically liver-enriched transcripts vital for establishing normal hepatic energy homeostasis, cell morphology and polarization. The hepatocytic differentiation is also accompanied by a reduction of the transcript content complexity.

## Abstract 2

Transcript profiling of HepaRG cells shows that translational regulation is the main genomic event associated with hepatocytic differentiation.

## Background

Liver diseases represent a major public health burden worldwide [1]. Upon acute liver injury, the mature hepatocytes demonstrate a major proliferative capacity. However, in chronic liver diseases such as chronic hepatitis B virus and hepatitis C virus infections and alcohol abuse, their regenerative potential is often impaired and liver progenitor cells, also called oval cells, significantly increase both in number and their capability to proliferate [2,3]. In recent years, liver progenitor cells have drawn special interest not only because of their regenerative capability and, therefore, therapeutic potential but also because of their possible contribution to liver carcinogenesis [4-6]. Rodent and simian liver progenitor cell lines have been established [7-10] and shown to successfully repopulate diseased livers [11-13].

The HepaRG cell line is a naturally immortalized human liver cell line with progenitor properties and bipotent differentiation-inducible capability that has been established from the non-tumoral region of a resected hepatitis C virus-associated hepatocellular carcinoma (HCC) [14,15]. These bipotent progenitor cells have been found to repopulate uPA/SCID mouse damaged livers [16]. Throughout differentiation, HepaRG cells evolve from a homogeneous dedifferentiated, depolarized, epithelial phenotype showing no specific organization to a structurally well-defined and polarized monolayer closely resembling those formed in primary human hepatocytes in culture, with canaliculi-like structures [15]. At the hepatocytic differentiated state, hepatocytic polarization markers such as ZO-1 and CD26 and liver-specific proteins such as albumin are expressed at levels similar to those found in normal liver biopsies [14,15]. Finally, iron storage and metabolism, typical features of mature normal hepatocytes, are intact in HepaRG cells [17]. Although this system bears limitations inherent to its pathological origin, it represents to date the only *in vitro *human model for hepatocytic differentiation.

We used this powerful system to identify the genomic events associated with the development of a functional and polarized hepatocyte-like cell from a previously dedifferentiated epithelial progenitor. A role for translational control in liver development and for translation regulators such as p70S6 kinase and 4E-BP1 upon liver regeneration has been previously reported [18-21]. Therefore, integrating polysome-bound RNA profiling to total RNA profiling not only provides highly relevant phenotypic information, but also provides insight into the role of translational control on the specific biological process studied.

## Results and discussion

### Total and polysome-bound RNA changes associated with hepatocytic differentiation of HepaRG cells

**Figure 1:**
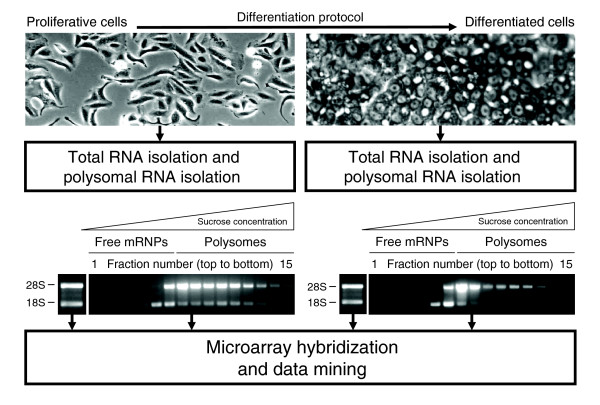
Pipeline for profiling of transcriptional and translational changes occurring during hepatocytic differentiation of HepaRG cells. Polysome fractions were identified as described in Materials and methods.

HepaRG cells were induced to differentiate into morphologically and functionally mature hepatocyte-like cells. Differentiated HepaRG cells showed features of normal hepatocytes, such as refractile cellular borders, clearly delineated nuclei and tridimensional polarization with the appearance of refringent circular canaliculi vertically (Figure 1). In order to identify the genomic events associated with HepaRG cell differentiation, total RNA and polysome-bound RNA were isolated at the proliferative stage and at the end of the differentiation protocol and analyzed on Affymetrix Human Genome U133A arrays (Figure 1). We separated polysomes from free messenger ribonucleoproteins (mRNPs) using sucrose gradient centrifugation with the assumption that translationally inactive mRNAs are present as free cytoplasmic mRNPs, whereas actively translated mRNAs are contained within polysomes. Total RNA was processed in parallel for each sample.

**Figure 2:**
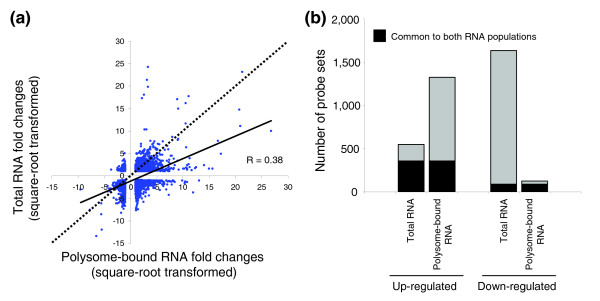
Correlation between total RNA and polysome-bound RNA fold changes upon HepaRG cell differentiation. **(a) **Plot drawn for the selected 3,071 probe sets between the square-root transformed polysome-bound RNA fold changes and the corresponding total RNA fold changes. The dotted line corresponds to a total/polysome-bound RNA ratio of 1 (slope = 1). The solid line is the regression curve calculated from all plots. **(b) **Number of probe sets regulated upon HepaRG cells differentiation. The number of up- or down-regulated probe sets upon differentiation were plotted against their RNA population of origin (either total RNA or polysome-bound RNA).

Out of the 22,283 probe sets spotted on the array, 3,071 (13.8%) were modulated by at least 2-fold upon differentiation and in 3 independent experiments, either in the total RNA or the polysome-bound RNA compartments. Total RNA fold changes were plotted against polysome-bound RNA fold changes for these 3,071 probe sets (Figure 2a). The correlation coefficient for the regression curve calculated from all values was 0.38, demonstrating a poor correlation and, therefore, an uncoupling phenomenon between changes in the polysome-bound fractions and changes in total RNA upon differentiation of HepaRG cells. We then determined the distribution of up- and down-regulated transcripts in each RNA population upon differentiation. In the total RNA compartment, 547 and 1,636 probe sets (a total of 2,183) were up-regulated and down-regulated, respectively. In contrast, in the polysome-bound RNA compartment, 1,325 and 124 probe sets (a total of 1,449) were up-regulated and down-regulated, respectively (Figure 2b). Transcription is, therefore, largely down-regulated during HepaRG differentiation while translation of specific genes is up-regulated. Probe sets that are similarly up-regulated or down-regulated in both RNA populations correspond to genes modulated as a result of transcriptional regulation without any subsequent translational control. These probe sets represented only a small number of genes with 359 up-regulated and 88 down-regulated probe sets. They represented 14.6% of the initially selected 3,071 regulated probe sets (Figure 2b, dark portions of the graph bars). On the other hand, 2,624 probe sets (85.4% of the total number of regulated probe sets) were modulated due to translational control (Figure 2b, gray portions of the bar graphs).

**Figure 3:**
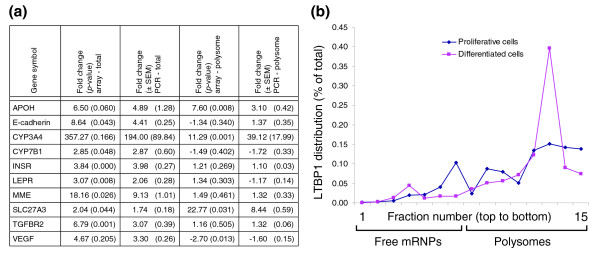
Validation of the array data by real time PCR **(a) **using total and polysome-bound RNA populations and **(b) **using individual fractions from mRNPs and polysomal fractions separated on sucrose gradient.

A subset of genes was selected for validation. Validation was performed using real-time PCR on the total RNA and the polysome-bound RNA populations, for ten genes: those encoding apolipoprotein H, solute carrier (SLC)27A3, cytochrome P450 isoforms 3A4 and 7B1, vascular endothelial growth factor (VEGF), E-cadherin, insulin receptor, leptin receptor, transforming growth factor (TGF) beta receptor 2 and membrane metallo-endopeptidase (MME). The PCR results obtained on the three independent experiments confirmed the microarray data for all ten genes (Figure 3a). Validation was also performed using real time PCR on each fraction of the sucrose gradient separating free mRNPs and polysomes, for three genes: those encoding latent transforming growth factor beta binding protein 1 (LTBP1), spectrin repeat-containing nuclear envelope 1 (SYNE-1) and matrix metalloproteinase 3 (MMP3). A shift was observed upon HepaRG differentiation for all three transcripts from the free mRNP fractions to the heavier polysome fractions on the sucrose gradient as shown in Figure 3b for LTBP1. These results demonstrate an increased translation of these transcripts and validate the array data indicating no change or a slight decrease in LTBP1, SYNE-1 and MMP3 transcript levels in the total RNA compartment and a strong up-regulation of all three transcripts in the polysome-bound RNA compartment.

All together, these results suggest that translational control plays a prominent role in the hepatocytic differentiation of liver progenitor cells and that the total RNA content may not be representative of the mature phenotype of hepatocyte-like cells. In addition, transcriptional changes did not overlap with translational changes. The large majority of polysome-bound (that is, translated) genes modified were up-regulated whereas the majority of genes modified at the total RNA level were down-regulated, suggesting that the mature hepatocyte phenotype is acquired by increased translation of pre-existing transcripts. The total RNA population can be considered as a stock of translated and untranslated transcripts that can be utilized by the cell rapidly. The more diverse the total RNA population is, the greater the options the cell has in selecting protein expression patterns. Therefore, the extensive down-regulation of genes in the total RNA compartment can be interpreted as a decrease in cellular RNA diversity, consistent with the commitment of a dedifferentiated epithelial progenitor into a defined, in this case hepatocytic, lineage.

### Polysome-bound RNA changes associated with HepaRG cell differentiation: the hepatocytic phenotype

To further characterize the differentiated phenotype of HepaRG cells, we selected all polysome-bound up-regulated probe sets (n= 1641) and all polysome-bound down-regulated probe sets (n= 204), regardless of their fold-change status at the total RNA level. The content of these two lists of genes were separately analyzed using the Ingenuity Systems Pathways Knowledge Base [22]. This database enables one to search for gene products' interactions and annotations coming from curated data from publications and peer-reviewed resources. Networks displaying significant overlap between the selected regulated genes found in our study and the software-preselected members were selected. The Ingenuity pathway analysis identified nine networks (networks A-I) and one network (network J) generated from the up-regulated and down-regulated transcripts, respectively (Table 1 and Additional data file 1). These ten networks can be divided into six groups based on their associated biological top functions: cell cycle, cell death, innate immunity, lipid and drug metabolism, cell morphology, and cell environment and movement.

**Table 1:** Biological networks and associated top functions generated from polysome-bound probe sets regulated upon HepaRG cell differentiation

Networks	Top functions	Members*
**Up-regulated**		
A	Cell cycle	ACTR2, C21ORF33, CAST, CCND3, CD86, CDC34, CKB, DACH1, EDA, FASTK, FKBP5, HDAC5, HSP90B1, **MEF2C**, **MEF2D**, **NF-KBIA**, PHB, PLCL1, PTMS, PTN, PTPN13, RAB5B, RAB5C, SF3B1, SF3B3, **SMARCA2**, **SMARCB1**, **SMARCC2**, TF, TMOD1, TSC22D3, UBE1
B	Cell death	ACO1, ACO2, ALB, ATRX, BCAP31, BRAF, CALR, **CASP8**, CFLAR, FCGRT, FOXA1, FTL, HLA-F, IFI16, **IGFBP1**, IHPK2, IL6R, **KNG1**, LRP1, MADD, MAP2K2, MDM2, NBN, NEK1, NOL3, PEBP1, RAD50, SIVA, THBS3, TSC2, TTR, ZNF350
C	Cell death Innate immunity	**BCL2**, **BCL2L11**, BCLAF1, BNIP3L, BSG, CAPN1, CAPN7, **CASP9**, CCNG2, DUSP6, **FOXO3A**, FRAT2, HBP1, **IRF3**, IRF7, LBP, MAP2, MAPT, MOAP1, NDRG1, NOSIP, PDCD8, PPP2R4, PTBP1, RARRES3, RBM5, SATB1, TEGT, TNFRSF11B, **TNFSF10**, TNFSF13, WWOX
D	Innate immunity	BBS4, **C3**, C1RL, C1S, **CDK5**, CDK5RAP2, CFB, CFI, DCTN1, DDB2, DHX9, ECM1, **ERBB3**, HP, IL6ST, MCM4, MCM5, NR3C2, OAS1, PCM1, PIAS1, PIN1, PIP5K1C, PPP1R1A, PTPN6, RASSF4 (includes EG:83937), RNF41, RRAS, SAP18, SERPING1, SP100, **STAT1**, TLN1
E	Lipid metabolism Drug metabolism	ADRA1A, AMPH, AP2A2, APBA3, **APOA1**, **APOC3**, BIN1, **CEBPD**, CPB2, DNM2, EFNA1, EHD1, EPPB9, FABP4, FGA, FGB, FGG, HELZ, HMGCS2, HSD17B4, IL13RA2, MECR, MLYCD, NCKIPSD, NR1H4, PLA2G2A, PLD1, **PPARA**, SMYD3, SORBS2, **STAT3**, SYT1, VAMP2, WASL
F	Lipid metabolism Drug metabolism	ACOX1, ADH6, BRD8, **CEBPA**, CEP350, CHI3L1, CRADD, CYP3A4, CYP3A5, CYP3A7, FABP1, GADD45G, H1FX, HADHA, HADHB, HPR, MPG, NFIL3, NR1H2, PCBP2, PEX11A, PLOD2, **PPARD**, **RXRA**, S100A8, S100A9, SERPINB1, SLC10A1, SMPDL3A, SULT2A1, TANK, UBN1
G	Lipid metabolism Drug metabolism	ACAA1, ACACB, ADH1A, ADH1B, ADH1C, ADM, **AGT**, AMACR, ATP1A1, CFH, DBP, DHCR7, EHHADH, FASN, FDPS, FXYD2, HLF, MEIS1, **MLXIPL**, MVD, MYH10, NSDHL, PEX5, PEX7, PPP1R12A, PURA, PYGL, RXRB, **SREBF1**, TCF8, TM7SF2, TXNIP, ZBTB16
H	Cell morphology	AIP, ANXA6, ARHGAP1, ARHGEF9, C13ORF24, **CBLB**, CD40, CDC42, COPA, COPB2, COPE, COPG, COPZ1, CUL5, DOCK9, DPP4, **FYN**, IQGAP1, **JAK2**, PDE4A, PIK3R1, PLCG1, PRMT5, PTPRA, SLIT2, SND1, SORBS1, **STAT6**, TCEB2, TIMP1, USP33
I	Cell environment	A2M, APOH, C5, C6, **EGR1**, ENPEP, F5, F10, **FN1**, IGFBP2, IL1R1, MAOB, MGP, **MMP3**, MTCP1, NAB2, NUP88, NUP214, NXF3, ORM1, SAPS2, SERPINA5, SERPINF2, SLC25A4, SOD2, SPARC, SPOCK3, ST6GAL1, TAOK2, TFPI, TFPI2, VPS45A, VTN
**Down-regulated**		
J	Cell movement	ACACA, BMP2, **CCL2**, **CSF1**, DDX21, FHL2, HGF, HNRPL, **IL8**, IRAK1, ITGA6, ITGAM, LIF, NCF2, PDGFB, POSTN, SERPINE1, SLC12A6, SYK, TGFB2, THBS1, TLR3, TNC, TNFAIP3, TRAF1, **VEGF**

*Members indicative of translational regulation are underlined. Members indicative of transcriptional regulation are not underlined. Members sharing the greatest number of connections within the network are in bold.

#### Cell cycle

Network A (Additional data file 1, A) was organized around transcription factors with tumor suppressor activities. These included three members of the SMARC tumor suppressor family (SMARCA2, SMARCB1 and SMARCC2), the transcription factors MEF2C and MEF2D and the NF-KB inhibitor NF-KB1A. Interestingly, several of these transcription factors (SMARC, MEF) remain uncharacterized in the liver.

#### Cell death

Network B (Additional data file 1, B) was associated with increased susceptibility to apoptosis and included the initiator caspase 8, insulin growth factor-binding protein (IGFBP)1, inhibitor of hepatocytic proliferation *in vivo *and *in vitro *[23], the interferon-induced gene IFI16, an essential mediator of p53 function [24] and tuberous sclerosis complex protein 2 (TSC2). The presence of Kininogen 1, a component of the coagulation cascade produced by the mature hepatocyte, confirmed the differentiation status of the cells. Cell death was also a top function of network C (Additional data file 1, C) with the presence of another member of the initiator caspase family, caspase 9, and of FOXO3A, known to trigger caspase 9-induced apoptosis. Other members associated with cell death included two strong inducers of apoptosis in human hepatocytes, TNFSF10/TRAIL [25] and IRF3 [26] and two members of the BCL2 family, BCL2 and BCL2L11. While BCL2 protects cells against apoptosis, BCL2L11 facilitates this process of cell death by neutralizing BCL2 antiapoptotic activity [27]. Therefore, the concomitant upregulation of BCL2 and BCL2L11, together with the pro-apoptotic genes described above, suggest that upon their differentiation, liver progenitor cells become highly susceptible to apoptosis. It has been reported that normal hepatocytes are highly sensitive to cell death upon, for example, drug-induced liver toxicity and that three-dimensional polarization, as occurs in this system (Figure 1), sensitizes hepatocytes to Fas apoptotic signaling [28]. Noteworthy, both up-regulated caspases identified (caspases 8 and 9) belong to the initiator caspase family, while none of the members of the effector caspase family (caspases 3, 6 and 7) [29] was affected, supporting the observation that the cells did not undergo apoptosis in culture.

#### Innate immunity

Another function associated with network C (Additional data file 1, C) was innate immunity and responses to viral infections, with the presence of two members of the interferon-regulatory factors, IRF3 and IRF7. IRF3 is a key component of innate immunity in the hepatocyte and has been shown to mediate interferon (IFN)β induction upon hepatitis C virus infection [30]. IRF7 is also mandatory for a proper IFNα-dependent antiviral response against hepatitis C virus [31]. Their up-regulation upon differentiation suggests an association between hepatocytic differentiation and innate immunity maturation. Maturation of the innate immunity upon differentiation was also suggested in network D (Additional data file 1, D) with the up-regulation of STAT1, one of the major components of the type I IFN transduction pathway, playing a key role in antiviral defense, inflammation and injury [32] and the up-regulation of complement C3 with a role in innate immunity as well as in acute phase response [33]. This network also included the EGFR-like receptor ERRB3 associated with cell survival and CDK5 reported to inhibit FAS/STAT3-dependent apoptosis in hepatoma cell lines *in vitro *and *in vivo *[34].

#### Lipid metabolism and drug metabolism

Network E (Additional data file 1, E) included the peroxisome proliferative activated receptor alpha (PPARA), regulating the expression of several hepatic genes and lipid homeostasis in the liver [35], as well as CEBPD and STAT3, key players in the control of the acute-phase response as well as in the protection of the hepatocyte upon acute phase-related injury [32,33,36]. As expected, apolipoproteins A1 and C3 as well as fibrinogens A, B, and G, markers of functional differentiation of the hepatocyte in relation to lipid metabolism and acute phase response, were strongly upregulated, downstream of PPARA, CEBPD and STAT3. Network F (Additional data file 1, F) included the liver-enriched transcription factors CAAT/enhancer-binding protein alpha (CEPBA), retinoid X receptor alpha (RXRA), and the peroxisome proliferative activated receptor delta (PPARD). CEBPA regulates two aspects of hepatic terminal differentiation: induction of differentiation-specific genes and repression of mitogenesis [37-39]. RXRA regulates cholesterol, fatty acid, bile acid, steroid, and xenobiotic metabolism and homeostasis in the liver. PPARD also plays a role in lipid metabolism, including cholesterol efflux and fatty acid oxidation [40,41], activates fat metabolism to prevent obesity [42], and regulates fatty acid synthesis, glucose metabolism and insulin sensitivity [43]. Network G (Additional data file 1, G) included the sterol regulatory element-binding transcription factor-1 (SREBF1), a major regulator of sterol biosynthesis, hepatic gluconeogenesis and lipogenesis in the liver [44], the liver-enriched transcription factor retinoid X receptor beta (RXRB) [45], MLXIPL, a glucose-responsive transcription factor that regulates carbohydrate metabolism in the liver [46], and angiotensinogen, an endocrine product of the hepatocyte regulating blood pressure [47]. ADH1A, ADH1B and ADH1C, mature hepatocyte-specific inducible genes involved in ethanol metabolism [48], were also included in this network.

#### Cell morphology

Network H (Additional data file 1, H) contained CDC42, a small GTPase involved in cell polarity. STAT6, also included in this network, is involved in the induction of a TH1 immune response to the hepatocyte and protects the normal parenchyma against liver injury [32]. Jak2 participates in transduction of interleukin (IL)6 signaling in case of acute phase reaction, as well as in the signal transduction of IFNγ [32]. The COP proteins (COPE, COPG, COPZ1, COPA, COPB2) mediate transport between the Golgi and the endoplasmic reticulum [49]. Their up-regulation may be associated with the increased flux of secreted proteins *en route *to the extracellular compartment through the Golgi complex after synthesis in the mature hepatocyte.

#### Cell environment and movement

Network I (Additional data file 1, I) included fibronectin (FN1), a co-factor of endogenous anti-angiogenic molecules and enhancer of cell attachment [50], and EGR1. EGR1 controls FIN1 and TGFβ1 gene expression and acts as a cell cycle blocker *in vitro *and *in vivo *through p53 [51]. This network also included MMP3, a secreted metalloprotease implicated in metastasis [52,53], IGFBP2, an insulin growth factor-binding protein associated with hepatocytic proliferation inhibition *in vivo *and *in vitro *[23] and two members of the serine protease inhibitors, SERPINF2 and SERPINA5. Network J (Additional data file 1, J), the only network associated with down-regulated polysome-bound probe sets, was also associated with cellular movement. Notably, the components of this network included several growth factors and secreted proteins implicated in angiogenesis and metastasis, such as hepatocyte growth factor (HGF), VEGF, platelet-derived growth factor (PDGF)-B, CCL2 and IL8. VEGF and PDGF-B are potent mitogenic and angiogenic factors [54]. HGF is the primary agent promoting the proliferation and apoptosis resistance of mature hepatocytes [55]. CCL2 is a monocyte chemoattractant [56]. IL8 is a proinflammatory cytokine and chemoattractant for neutrophils [57]. Therefore, differentiation of hepatocytic progenitors seems to be associated with a progressive disappearance of an inflammation-like state, as shown by the down-regulation of several chemoattractants and proinflammatory messengers.

Taken together, this analysis identified the regulation of functions specific to a differentiated hepatocytic phenotype. Up-regulation of transcripts belonging to the well known liver-enriched transcription factors, such as CEBPA, RXRA, RXRB, and PPARD, as well as down-regulation of NF-KB expression, are correlated with the differentiation of liver progenitor cells into morphologically and functionally mature hepatocyte-like cells. This study also revealed the involvement of lesser known nuclear proteins in the hepatocytic biology, such as SMARC, MEF and EGR1 proteins, and novel associations, such as the role of several IFN-associated or induced proteins in the acquisition of the hepatocytic phenotype. STAT1 is one of the key elements for the induction of the type I IFN response. Its up-regulation, as well as the up-regulation of several other IFN-related transcripts (OAS1, IRF3, IRF7 and IFI16), suggest that acquisition of key elements to innate immunity is associated with hepatocytic differentiation. It would be interesting, therefore, to investigate if the progenitor cell compartment in regenerative livers of chronically hepatitis B or C virus-infected patients is more prone to viral replication because of an immature innate immunity status.

### Contribution from translation

**Figure 4:**
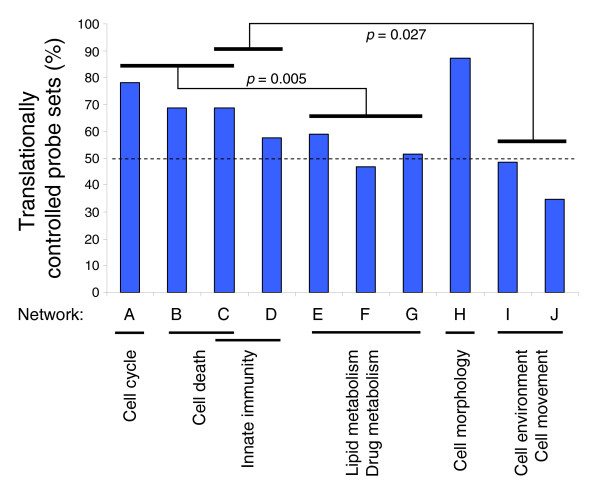
Translational control associated with hepatocytic differentiation targets specific cellular functions. Percentages of translationally regulated probe sets in a given network were calculated for all networks generated from the regulated probe sets identified in the polysome-bound RNA population (networks A to J depicted in Additional data file 1 and listed in Table 1). Paired *t*-tests were performed between groups of networks associated with distinct biological functions and significant *p-*values (*p *< 0.05) are indicated. The dashed line indicates 50% of translationally regulated probe sets.

Most of the genes identified in this study and contributing to the differentiation phenotype were modulated by translational control. Translationally regulated transcripts are underlined in Table 1 and indicated in blue in Additional data file 1. To investigate whether translational control specifically affects transcripts involved in defined cellular functions, we calculated the percentage of translationally controlled probe sets in each of the ten networks A-J described above. Paired *t*-tests were performed between groups of networks sharing the same cellular functions (Figure 4). A significantly greater involvement of translational control was observed in networks related to cell cycle and cell death functions than in networks related to lipid metabolism and drug metabolism (*p *= 0.005). Likewise, a significantly stronger involvement of translational control was found in innate immunity-related networks compared to cell environment and cell movement-related networks (*p *= 0.027). The high percentage of translationally controlled probe sets in cell cycle and cell death-related networks is in agreement with the ability of the hepatocyte to massively and rapidly proliferate under acute liver injury, as well as with the hypersensitivity of the hepatocyte to cell death in response, for example, to drug-associated toxicity. Translationally regulated transcripts associated with cell cycle included the nuclear proteins SMARCA2 and SMARCB1, the transcription factors MEF2C, MEF2D and EGR1 and the NF-KB inhibitor NFKBIA. Translationally regulated transcripts associated with cell death included oncostatin M receptor/IL6ST and the initiator caspases 8 and 9. Translationally regulated transcripts associated with innate immunity included several interferon-associated genes, such as those encoding OAS1, IRF3 and IFI16. Finally, numerous transcription factors associated with inflammation were translationally upregulated and included the three liver-enriched transcription factors RARA, RXRA and RXRB and STAT6 (Table 2).

**Table 2:** Selected transcripts

	Total (fold change)	*p-*value	Polysome (fold change)	*p-*value
**Contribution from translation**				
SMARCA2	+ 1.46	NS	+ 4.26	0.009
SMARCB1	- 4.00	NS	+ 4.36	0.024
MEF2C	+ 1.31	NS	+ 3.98	0.004
MEF2D	- 1.29	NS	+ 2.35	0.018
NF-KBIA	+ 1.24	NS	+ 2.81	0.027
Oncostatin M receptor/IL6ST	+ 1.24	NS	+ 80.12	0.010
Caspase 8	- 1.92	NS	+ 4.17	0.010
Caspase 9	+ 1.26	NS	+ 3.50	0.025
OAS1	- 1.69	NS	+ 4.82	0.016
IRF3	+ 1.00	NS	+ 5.65	0.034
IFI16	+ 1.90	NS	+ 54.11	0.009
RARA	- 1.19	NS	+ 2.69	0.050
RXRA	+ 1.45	NS	+ 2.17	0.004
RXRB	- 1.23	NS	+ 4.45	0.013
STAT6	+ 1.29	NS	+ 2.99	0.011
EGR1	+ 1.52	NS	+ 12.34	0.050
IGFBP1	+ 1.87	NS	+ 7.38	0.010
MMP3	- 1.10	0.044	+ 6.87	0.047
SLC27A3	+ 2.03	0.043	+ 22.77	0.031
				
**Contribution from transcription**				
PPARA	+ 2.25	0.002	+ 2.49	0.026
PPARD	+ 2.00	0.001	+ 3.80	0.005
CEBPA	+ 3.91	0.050	+ 3.86	0.004
HLF	+ 14.11	0.001	+ 17.09	0.015
ADH1B	+ 354.89	0.021	+ 335.41	0.050
ADH1C	+ 38.86	0.050	+ 27.37	0.003
ADH6	+ 18.22	0.029	+ 46.55	0.050
ApoH	+ 6.49	0.050	+ 7.59	0.008
SERPINA1	+ 2.70	0.007	+ 10.69	0.015
SERPINA4	+ 4.86	0.050	+ 18.58	0.037
SERPINF2	+ 9.82	0.050	+ 2.30	0.000
Complement component 1, s	+ 2.30	0.008	+ 3.40	0.041
Complement component 3	+ 2.28	0.050	+ 2.98	0.042
Complement component 4A	+ 6.65	0.006	+ 7.86	0.025
Complement component 5	+ 3.42	0.039	+ 2.33	0.014
Complement component 6	+ 36.20	0.000	+ 48.42	0.043
Fibrinogen A	+ 15.35	0.017	+ 9.84	0.022
Fibrinogen B	+ 17.13	0.033	+ 15.02	0.033
Fibrinogen G	+ 14.79	0.015	+ 11.55	0.016
TNFSF10/TRAIL	+ 7.08	0.002	+ 5.15	0.007
IL6R	+ 9.30	0.000	+ 15.95	0.012
BMP2	- 2.08	0.000	- 2.00	0.014
PDGFB	- 2.53	0.013	- 2.62	0.003
				
**Translational repression**				
MME	+ 18.16	0.025	+ 1.48	NS
E-cadherin	+ 8.64	0.043	- 1.35	NS
CYP3A4	+ 357.26	NS	+ 11.29	0.015
CYP3A5	+ 20.14	0.012	+ 4.66	NS
CYP2B6	+ 22.78	0.021	+ 2.55	0.016
CYP7B1	+ 2.85	0.048	- 1.49	NS
CYP2A6	+ 2.85	0.002	- 1.51	NS
CYP2C19	+ 17.70	NS	+ 1.35	0.032
CYP4F3	+ 19.58	0.038	+ 5.14	NS
TGFBR2	+ 6.78	0.001	+ 1.15	NS
VEGF	+ 4.67	0.034	- 2.70	NS
Insulin receptor	+ 3.83	<0.001	+ 1.20	NS
Leptin receptor	+ 3.07	0.007	+ 1.34	NS

NS, not significant.

Numerous transcription factors were translationally upregulated while left unchanged or even decreased at the total RNA level. Translational control of these transcription factors provides the cell with a means to modify its phenotype in a timely manner, rapidly expressing genes downstream of these transcription factors. The hepatocyte has to be a highly versatile cell because of at least two of its functions: the ability to generate the acute phase reaction and to maintain blood homeostasy after meals as the first line organ downstream of the portal vein that carries nutrients from the digestive tract.

The importance of translational control during liver progenitor cell differentiation raises the question of the identity of the actors involved. We recently reported a functional down-regulation of the mTOR/4E-BP1/p70S6 kinase pathway during differentiation of HepaRG cells [58]. Moreover, forced expression of an activated mutant of mTOR impairs hepatocytic differentiation in this model [58]. This pathway may therefore contribute at least partially to some of the translational events described here.

### Contribution from transcription

Some genes were similarly modified upon differentiation of HepaRG cells, in both the total and the polysome-bound RNA populations, indicative of a transcriptional regulation. These include 435 up-regulated and 142 down-regulated probe sets (Figure 2b), indicated in yellow in Additional data file 1 and not underlined in Table 1. These genes corresponded in the majority to liver-enriched transcripts and to genes involved in lipid and drug metabolism. They included those encoding PPARA, PPARD, CEBPA, the hepatic leukemia factor (HLF) and the alcohol dehydrogenases 1B, 1C and 6. Other transcriptionally regulated genes included those encoding plasma proteins synthesized in the liver: the SERPINs A1, A4, F2, several complement system subunits (C1S, C3, C4A, C5 and C6) and three forms of fibrinogen (A, B and G). Finally, several cytokines, chemokines or hormones and their receptors were transcriptionally regulated as well: TNFSF10/TRAIL, IL6R, BMP2 and PDGFB (Table 2).

**Figure 5:**
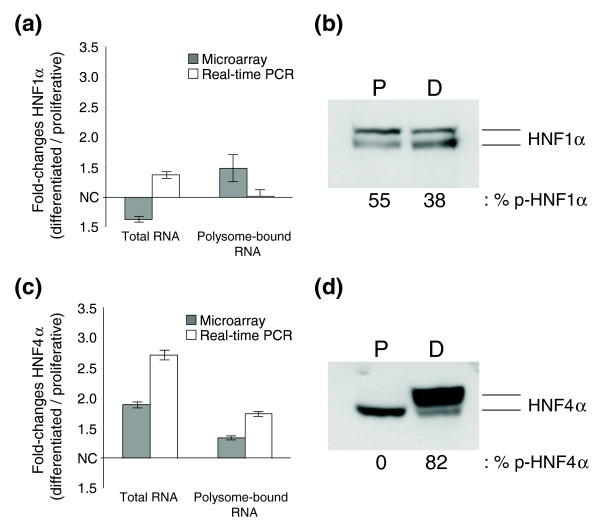
Transcriptional, translational, and post-translational regulation of HNF1α and HNF4α during HepaRG cell differentiation. **(a,c) **Modulation of HNF1α (a) and HNF4α (c) in total and polysome-bound RNA populations throughout differentiation, assessed by microarray and by quantitative PCR. For microarray data, values and error bars correspond to the mean ± SEM of three independent differentiation experiments. For real-time PCR data, values and error bars correspond to the mean ± SEM of three independent measures. **(b,d) **Protein expression levels and phosphorylation status of HNF1α (b) and HNF4α (d) in proliferative (P) and differentiated (D) cells. The percentage of the phosphorylated forms is indicated. Results are representative of three independent differentiation processes.

As the contribution of transcription appeared restricted to selective genes during HepaRG cell differentiation, we sought to investigate the expression levels and phosphorylation status of the canonic hepatocytic transcription factors HNF1α and HNF4α throughout differentiation. HNF1α is a major player in the acquisition of central hepatocytic functions, including gluconeogenesis, carbohydrate synthesis and storage, lipid metabolism (synthesis of cholesterol and apolipoproteins), detoxification (synthesis of cytochrome P450 monooxygenases), and synthesis of serum proteins (albumin, complements, and coagulation factors) [59]. Interestingly, neither total nor polysome-bound RNA levels of HNF1α were modulated (-1.38 and +1.48-fold, respectively). This observation was confirmed by real time PCR (+1.38 ± 0.08 fold (mean ± standard error of the mean (SEM)) in total RNA and +1.02 ± 0.19 fold (mean ± SEM) in polysome-bound RNA; Figure 5a). In addition, no changes were observed at the protein expression level nor in phosphorylation status for HNF1α (55% of HNF1α is phosphorylated at the proliferative stage versus 38% at the differentiated stage; Figure 5b). HNF4α was slightly increased in both total and polysome-bound RNA (+1.89-fold and +1.35-fold, respectively). These slight increases were confirmed by real time PCR (+2.71 ± 0.13 fold (mean ± SEM) in total RNA and +1.74 ± 0.06 fold (mean ± SEM) in polysome-bound RNA; Figure 5c). However, HNF4α phosphorylation was strongly induced upon differentiation (Figure 5d), suggesting that, in contrast to HNF1α, HNF4α may contribute to HepaRG cell differentiation. Mutations of HNF1α associated with metabolic diseases have been described [60,61] and, therefore, we cannot exclude that the lack of regulation of HNF1α found in this study results from mutation(s) disrupting its biochemical characteristics. However, the patient that gave rise to HepaRG cells was not known to be affected by any of these diseases.

In conclusion, transcriptional control appears to play a highly selective role in the phenotype of liver progenitor cell maturation and specifically targets liver-enriched transcripts characteristic of the mature hepatocytic phenotype. Novel findings suggest that the complement system is induced during maturation following transcriptional regulation.

### Translational repression

Several transcripts were strongly transcriptionally induced upon HepaRG cell differentiation while unchanged or induced to a much weaker level in the polysome-bound RNA population, suggesting a translational repression control. Examples include E-cadherin, involved in hepatocytic polarization, cytochrome P450 3A4, a steroid-inducible cytochrome P450 isoform, cytochrome P450 7B1, a cytochrome P450 isoform involved in cholesterol metabolism, cytochrome P450 2A6 and 2C19, cytochrome P450 isoforms involved in drug metabolism, TGF-β receptor 2 and VEGF, an important regulator of angiogenesis and metastasis (Table 2).

Interestingly, four isoforms of cytochrome P450 were strongly up-regulated at the total RNA level but not at the polysome-bound RNA level. Given that cytochromes are inducible proteins involved in drug and lipid metabolism, high levels of untranslated RNA could serve as a stock that may be rapidly translated and used for the detoxification and acute phase-associated functions of the hepatocyte.

## Conclusion

The most prominent result of this study is a strong association between translational control and hepatocytic differentiation of liver progenitor cells, as demonstrated by the fact that the great majority of the regulated genes have been identified in the polysome-bound RNA population and not in the total RNA population. Another interesting feature supporting the involvement of translational control in hepatocytic differentiation of liver progenitor cells is that the large majority of polysome-bound transcripts modified upon differentiation were up-regulated whereas the majority of genes modified in the total RNA population were down-regulated. Altogether, these data suggest that the mature hepatocyte phenotype is acquired by increased translation of pre-existing transcripts and is associated with a reduction in the diversity of transcripts that the differentiated cell can utilize, consistent with the commitment of a dedifferentiated epithelial progenitor into a defined hepatocytic lineage. This study increases our knowledge on gene expression regulation of liver progenitor cells upon differentiation, providing novel paths to successfully use liver progenitor cells to repopulate diseased livers.

## Materials and methods

### Cell culture

The HepaRG cell line was cultured in William's E medium (Invitrogen, Carlsbad, CA, USA) supplemented with 10% fetal calf serum (Mediatech, Manassas, VA, USA), 100 units/ml penicillin, 100 μg/ml streptomycin (Invitrogen), 5 μg/ml insulin (Sigma-Aldrich, St. Louis, MO, USA), and 5 × 10^-5 ^M hydrocortisone hemisuccinate (Sigma-Aldrich). To induce differentiation, a two-step procedure was used as previously described [14,15]. Cells were seeded at a density of 4 × 10^4 ^cells/cm^2 ^and maintained for 2 weeks in the growth medium. Then, the culture medium was supplemented with 1% DMSO (Sigma-Aldrich) and 20 ng/ml EGF (PeproTech, Rocky Hill, NJ, USA) for 2 additional weeks. Cells were harvested either at 2 days (proliferative stage) or at 28 days (differentiation stage) after seeding. Cell culture pictures were taken using a phase contrast microscope (Nikon). Differentiation was evaluated morphologically by counting bile canaliculi (refringent area) at the intersection of two or three hepatocyte-like cells.

### Total RNA extraction and polysome fractionation

Total RNA was extracted, precipitated and resuspended in RNAse-free water using Trizol reagent (Invitrogen) according to the manufacturer's instructions. For polysome fractionation, cycloheximide (100 μg/ml) was added to the medium for 3 minutes prior to harvest. The medium was then removed and the cells were washed with ice-cold phosphate-buffered saline containing 100 μg/ml cycloheximide. The cells were then scraped, centrifuged at 800g for 5 minutes at 4°C and cytoplasmic RNA was obtained by lysis of the cell pellet in 1 ml of polysome buffer containing 10 mM Tris-HCl (pH 8.0), 140 mM NaCl, 1.5 mM MgCl2, 0.5% Nonidet P-40, and a ribonuclease inhibitor, RNasin (500 units/ml; Promega, Madison, WI, USA). After the removal of nuclei, the cytosolic supernatant was supplemented with 100 μg/ml cycloheximide, 665 μg/ml heparin, 20 mM dithiothreitol, and 1 mM phenylmethanesulfonyl fluoride. Mitochondria and membrane debris were removed by centrifugation, and the post-mitochondrial supernatant was overlaid onto a 15-40% sucrose gradient and spun at 38,000 rpm for 2 h at 4°C in a SW41Ti rotor (Beckman Coulter, Fullerton, CA, USA). Fractions (750 μl) were collected from the top of each gradient and deproteinated with 100 μg of proteinase K in the presence of 1% SDS and 10 mM EDTA. After acid phenol extraction, RNA integrity was controlled by electrophoresis analysis on 1.2% agarose gel. Densitometry (GelDoc, Bio-Rad Laboratories, Hercules, CA, USA) was used to identify the fractions in which the 28S/18S ratio equals 2 (that is, fractions corresponding to polysome-bound RNA). These fractions were pooled from each sucrose gradient.

### Microarray hybridization and data mining

Total and polysome-bound RNAs were purified using the RNeasy mini-kit clean-up protocol (Qiagen, Valencia, CA, USA), RW1 buffer being used to efficiently remove heparin from the samples. The first-strand cDNA, the double-strand cDNA, and cRNA were synthesized, and cRNA was fragmented using Affymetrix kits and guidelines [62]. All cRNA final products were tested in terms of amount and integrity by Bioanalyzer (Agilent Technologies, Santa Clara, CA, USA) prior to microarray hybridization. cRNA samples were processed on Affymetrix HGU133A arrays with strict adherence to the labeling, hybridization and staining protocols provided by Affymetrix. A 'present' (P), 'marginal' (M) or 'absent' (A) call was assigned to each probe set using Affymetrix GeneChip Operation Software (GCOS v1.4). Probe sets with an 'absent' (A) call on all arrays were filtered out. Background correction and normalization steps were carried out using the GC-RMA method and the R Bioconductor software [63]. Microarray data have been deposited in the ArrayExpress repository [64] under the accession number E-MEXP-1082. Three independent differentiation processes were performed and the correlation coefficients between each duplicate at the proliferative and the differentiated level were calculated using scatter plots. For total RNA, the correlation coefficients were 0.98 and 0.95 for proliferative and differentiated cells, respectively. For polysome-bound RNA, the values were 0.98 and 0.97 for proliferative and differentiated cells, respectively. The Ingenuity pathway analysis [22] was used to analyze selected probe sets. Each gene identifier was mapped to its corresponding gene object in the Ingenuity Pathways Knowledge Base. The application utilizes a right-sided Fisher's exact test to identify networks that had higher odds ratio of containing significant genes. These genes, called Focus Genes, were then overlaid onto a global molecular network. Networks of these Focus Genes were then algorithmically generated.

### Western-blotting

Cells were lysed in 50 mM Tris-HCl (pH 8), 150 mM NaCl, 0.1% SDS, 1% NP-40 supplemented with protease inhibitors (Complete, Roche Diagnostics, Indianapolis, IN, USA). Thirty micrograms of proteins were resolved on 10% SDS-polyacrylamide gels and electrotransferred onto nitrocellulose membrane (Amersham Biosciences, Piscataway, NJ, USA). Equal loadings and homogeneous blotting were confirmed using Ponceau red staining. Membranes were blocked with 5% nonfat milk in Tris-buffered saline and incubated with primary antibodies (anti-HNF1α and anti-HNF4α, Santa Cruz Biotechnology, dilution 1/500, Santa Cruz, CA, USA) overnight. Horseradish peroxidase-conjugated immunoglobulins (Dako, dilution 1/1,000, Carpinteria, CA, USA) were used as secondary antibodies and proteins were visualized with enhanced ECL chemiluminescent reagent (Amersham Biosciences). Densitometry was performed using the Total Lab TL100 software.

### Real-time PCR

One microgram of DNAse I-treated (Promega) total RNA or polysome-bound RNA was reverse transcribed using Moloney murine leukemia virus reverse transcriptase and random hexamers (Invitrogen) for 50 minutes at 42°C. cDNA mixtures (1/10) were mixed with an equal volume of 2 × iQ SYBR green supermix (Bio-Rad Laboratories). Amplification was then performed at an annealing temperature of 55°C or 60°C and an elongation time of 30 s or 1 minute on a MyIQ real-time PCR apparatus (Bio-Rad Laboratories). Primer sequences were obtained through the Primer Bank website [65,66] and are described in Additional data file 2. Differential expression ratios between proliferative and differentiated stages were calculated using the Δ(ΔCt) formula. Specificity of all amplicons was assessed by post-run melting curve analysis and agarose gel electrophoresis. For the analysis on sucrose gradient fraction distribution, 100 μl of each fraction harvested from the sucrose gradients were purified using the RNAeasy kit (Qiagen). Ten microliters of collected RNA were DNAse-digested and reverse transcribed as described above. Since the RNA amount in each fraction was different, in order to avoid efficiency differences of the reverse transcriptase, RNA amounts were equalized by adding an appropriate amount of *in vitro *transcribed irrelevant RNA to each fraction, giving a final amount of 1 μg of RNA on each sample. One tenth of each cDNA reaction was processed by real-time PCR as described above.

## Abbreviations

CEPBA, CAAT/enhancer-binding protein alpha; FN1, fibronectin; HGF, hepatocyte growth factor; HLF, hepatic leukemia factor; IFN, interferon; IGFBP, insulin growth factor-binding protein; IL, interleukin; LTBP1, latent transforming growth factor beta binding protein 1; MME, membrane metallo-endopeptidase; MMP3, matrix metalloproteinase 3; mRNP, messenger ribonucleoprotein; PDGF, platelet-derived growth factor; PPARA, peroxisome proliferative activated receptor alpha; PPARD, peroxisome proliferative activated receptor delta; RXRA, retinoid X receptor alpha; RXRB, retinoid X receptor beta; SEM, standard error of the mean; SERPIN, serine protease inhibitor; SLC, solute carrier; SREBF1, sterol regulatory element-binding transcription factor-1; SYNE-1, spectrin repeat-containing nuclear envelope 1; TGF, transforming growth factor; TSC2, tuberous sclerosis complex protein 2; VEGF, vascular endothelial growth factor.

## Authors' contributions

RP carried out the study, participated in its design and drafted the manuscript. LB conceived the study and finalized the manuscript. Both authors read and approved the final manuscript.

## Additional data files

The following additional data are available. Additional data file 1 is a figure showing the polysome-bound generated networks associated with differentiation of HepaRG cells. Additional data file 2 is a table listing the primer sequences and the lengths of the associated amplicons.

## Supplementary Material

Additional data file 1 Ten networks were identified by the Ingenuity pathway analysis: nine networks (networks A-I) generated from the up-regulated transcripts and one network (network J) generated from the down-regulated transcripts (see also Table 1). Networks were generated from all polysome-bound regulated probe sets upon differentiation of HepaRG cells and classified according to their respective biological top functions. Networks are represented as nodes displayed using various shapes that represent the functional class of the gene product and lines/arrows displayed with various labels that describe the specific relationship between the nodes. Translationally and transcriptionally controlled transcripts are shown in blue and in yellow, respectively. Gene abbreviations are located within the symbol. Solid and dotted lines depict direct and indirect interactions, respectively. An asterisk appears next to any gene for which the input file contained more than one identifier; in that case, the maximum value is displayed. A, activation/deactivation; RB, regulation of binding; PR, protein-mRNA binding; PP, protein-protein binding; E, expression; I, inhibition; L, proteolysis M, biochemical modification; P, phosphorylation/dephosphorylation; T, transcription; LO, localization. Click here for file

Additional data file 2 Primer sequences and the lengths of the associated amplicons. Click here for file
