# Supplementary figures and images for: Translational control plays a prominent role in the hepatocytic differentiation of HepaRG liver progenitor cells
Source: Genome Biol. 2008 Jan 25;9(1):R19. doi: 10.1186/gb-2008-9-1-r19 (PMC2395229; doi:10.1186/gb-2008-9-1-r19)

## Cell death

# B

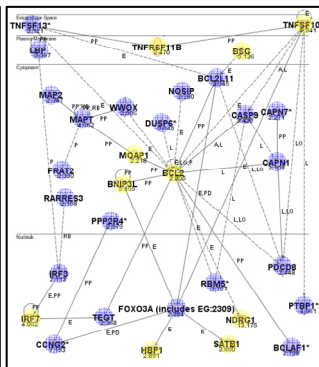

**C**

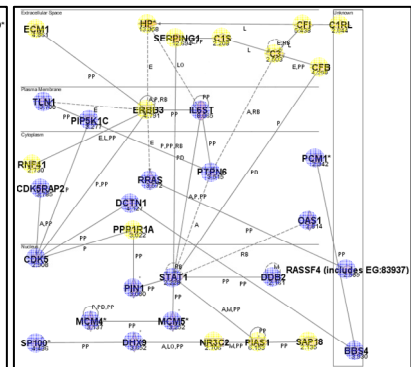

D

# E

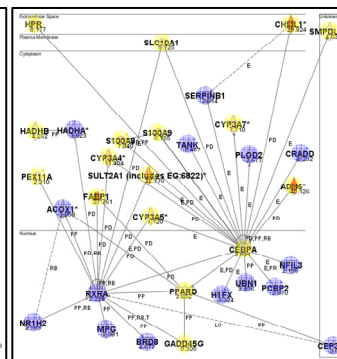**F**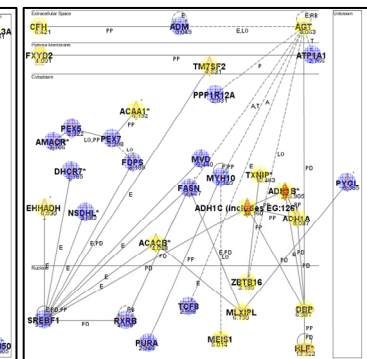

**G**

H

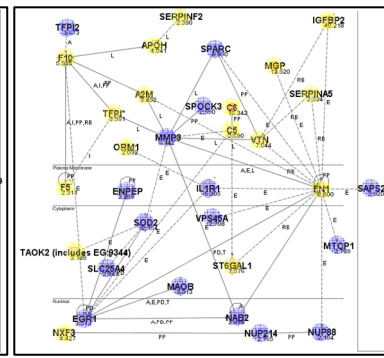

!

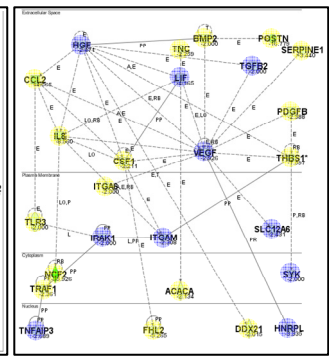

J

Supplement: Additional data file 1 — Ten networks were identified by the Ingenuity pathway analysis: nine networks (networks A-I) generated from the up-regulated transcripts and one network (network J) generated from the down-regulated transcripts (see also Table 1). Networks were generated from all polysome-bound regulated probe sets upon differentiation of HepaRG cells and classified according to their respective biological top functions. Networks are represented as nodes displayed using various shapes that represent the functional class of the gene product and lines/arrows displayed with various labels that describe the specific relationship between the nodes. Translationally and transcriptionally controlled transcripts are shown in blue and in yellow, respectively. Gene abbreviations are located within the symbol. Solid and dotted lines depict direct and indirect interactions, respectively. An asterisk appears next to any gene for which the input file contained more than one identifier; in that case, the maximum value is displayed. A, activation/deactivation; RB, regulation of binding; PR, protein-mRNA binding; PP, protein-protein binding; E, expression; I, inhibition; L, proteolysis M, biochemical modification; P, phosphorylation/dephosphorylation; T, transcription; LO, localization. [file gb-2008-9-1-r19-S1.pdf]
